# Severity and Cost of Respiratory Syncytial Virus Versus Influenza in Hospitalized Adults in Spain

**DOI:** 10.1111/irv.70254

**Published:** 2026-04-22

**Authors:** Elisa Correcher‐Martínez, Cintia Muñoz‐Quiles, Mónica López‐Lacort, Ainara Mira‐Iglesias, María Díaz‐López, Beatriz Mengual‐Chuliá, F. Xavier López‐Labrador, Javier Díez‐Domingo, Alejandro Orrico‐Sánchez

**Affiliations:** ^1^ Vaccines Research Unit. Fundación para el Fomento de la Investigación Sanitaria y Biomédica de la Comunitat Valenciana FISABIO‐Public Health Valencia Spain; ^2^ CIBER de Epidemiología y Salud Pública Instituto de Salud Carlos III Madrid Spain; ^3^ Virology Laboratory, Genomics and Health Area, Fundación para el Fomento de la Investigación Sanitaria y Biomédica de la Comunitat Valenciana FISABIO‐Public Health Valencia Spain; ^4^ Universidad Católica de Valencia San Vicente Mártir Valencia Spain

**Keywords:** adults, hospital mortality, hospitalization, influenza, length of stay, monitoring, prospective, respiratory syncytial virus, retrospective, reverse transcriptase polymerase chain reaction

## Abstract

**Background:**

Respiratory syncytial virus (RSV) can lead to severe outcomes, particularly in older and vulnerable adults, yet it remains underdiagnosed due to limited testing. This study compared the severity and costs of laboratory‐confirmed RSV versus influenza hospitalizations.

**Methods:**

We performed a retrospective analysis using data from the Valencia Hospital Network for the Study of Infectious Diseases (VAHNSI), covering up to 46% of the Valencia Region's population. Hospitalized influenza‐like illness (ILI) cases in individuals aged ≥ 65 years across six seasons (2014/15–2019/20) were included. RSV and influenza were identified by multiplex RT‐PCR. Outcomes assessed included mechanical ventilation (MV), hospital length of stay (LOS), in‐hospital death, 30‐day post‐discharge death, and hospitalization costs. Logistic and log‐normal regression models were used to compare severity and cost outcomes between RSV and influenza, adjusting for age, sex, comorbidities, and influenza vaccination status. Odds ratios (ORs) and mean ratios (MRs) with 95% confidence intervals (CIs) were calculated.

**Results:**

Of 1954 hospitalizations, 429 (22%) were due to RSV and 1525 (78%) to influenza. RSV cases showed higher MV use (9.26% vs. 4.15%; adjusted OR 2.43 [95% CI: 1.61–3.67]) and longer LOS (7.7 vs. 6.8 days; adjusted MR 1.10 [95% CI: 1.02–1.19]). Mortality outcomes were similar. RSV hospitalizations incurred 6% higher mean costs (€3907 vs. €3806).

**Conclusions:**

RSV hospitalizations in older adults were less frequent but more severe and costly than influenza, with comparable mortality. These findings highlight the importance of RSV prevention and support the development of targeted vaccination programs for the elderly.

## 1 Introduction

Respiratory syncytial virus (RSV) is a well‐established cause of acute respiratory infections (ARI) in young children [1], but growing evidence highlights its significant and often under‐recognized impact on older and vulnerable adults [2, 3, 4, 5, 6, 7, 8, 9]. With the recent approval of the first RSV vaccines for older populations in the USA and Europe [10, 11, 12], and more candidates under development [13], accurately characterizing the burden and severity of RSV in these groups has become a pressing public health priority to establish immunization strategies and to evaluate their impact.

In contrast, influenza infections have been extensively studied across diverse populations for decades. Comparing RSV and influenza epidemiology may help raise awareness of RSV's impact on older adults. Several studies have attempted this, yielding mixed results [2, 14, 15, 16, 17, 18, 19, 20]. While many studies indicate that Intensive Care Unit (ICU) admission, mechanical ventilation (MV), hospital length of stay (LOS), and case fatality rates are comparable or even higher in RSV‐related hospitalizations than in influenza cases [2, 14, 15, 16, 21], others have reported greater severity with influenza [19, 20]. However, most studies are limited to one or a few seasons, or a single hospital, reducing sample sizes and limiting the reliability of estimates [16, 18, 19, 20, 22].

Moreover, influenza vaccination has been shown to attenuate disease severity in breakthrough infections, with studies reporting fewer ICU admissions, lower mortality, and milder symptoms, such as fever, in vaccinated patients compared with unvaccinated [23]. However, no studies have considered influenza vaccination status when comparing the severity of RSV and influenza hospitalizations during the pre‐pandemic period. The few data published on the post‐pandemic period [17, 18] found that RSV severity was comparable with unvaccinated influenza cases, but substantially higher than in vaccinated influenza patients.

Similarly, while the economic burden of influenza is well‐documented, particularly among older adults and high‐risk groups, it remains underexplored in the case of RSV [24]. Some data suggest that per‐episode healthcare costs are comparable between the two viruses [25, 26]. In Spain, only one retrospective observational cohort study has compared the hospitalization costs of RSV and influenza using a nationwide database [26].

RSV comprises two antigenically distinct subtypes, A and B, which often co‐circulate. Although both can cause infections of similar severity, several studies suggest that RSV‐A may be associated with increased transmissibility and greater disease severity in young children [27, 28]. However, there is limited prior research comparing clinical severity between RSV genotypes A and B in older adults [18, 29, 30, 31]. Understanding subtype‐specific epidemiology will be critical for accurately evaluating the impact of interventions such as new RSV vaccines and monoclonal antibodies.

To address these gaps, we compared severity outcomes and hospitalization costs associated with RSV and influenza, accounting for influenza vaccination status. We also analyzed clinical severity differences between RSV subtypes A and B. Using data from six pre‐pandemic seasons (2014/15–2019/20) collected through the Valencia Hospital Network for the Study of Infectious Diseases (VAHNSI) hospital active surveillance network in Spain [9, 32, 33, 34], and linked to the comprehensive Valencia Health System Integrated Database (VID) [35], we conducted the first direct comparison of RSV and influenza hospitalizations in Spanish adults based on prospectively collected, population‐based data over a long pre‐pandemic period.

## 2 Methods

### 2.1 Study Design and Setting

A retrospective study was conducted using data from VAHNSI and VID.

VAHNSI is an active surveillance network in place since 2010, prospectively analyzing respiratory hospitalizations in tertiary‐care public hospitals in the Valencia Region of Spain. The study is conducted in 4–10 hospitals (depending on the season; Appendix S1) covering 21%–46% of total inhabitants of the Valencia Region of Spain (around 5 M). In VAHNSI, from Monday to Saturday, full‐dedicated researchers systematically screen and contact patients of all age groups admitted through the Emergency Department within the preceding 8–48 h, with a diagnosis potentially related to respiratory infections. Noninstitutionalized subjects residing within the catchment area of the VAHNSI participating hospitals for at least 6 months, without any prior hospital admissions in the last 30 days (to avoid capturing nosocomial infections and readmissions related to a previous event), and who provide written consent (or had it provided by a legal representative) are systematically interviewed. Influenza and RSV testing is performed on all included subjects by multiplex real‐time reverse transcription‐polymerase chain reaction (RT‐PCR) [9, 36].

VID is a set of multiple public, population‐wide electronic health record databases [35], which provides exhaustive longitudinal information including sociodemographic data, clinical, pharmaceutical (prescription, dispensation), immunization status, and healthcare utilization data from hospital care, emergency departments, specialized care, primary care, and other public health services. Each individual has a personal identification number that allows for linking all variables at the individual level from VAHNSI and VID.

Severity and mortality outcomes, as well as hospitalization costs, were compared between patients hospitalized with laboratory‐confirmed RSV and those hospitalized with laboratory‐confirmed influenza. A similar comparison was conducted between patients hospitalized with RSV A and those hospitalized with RSV B.

### 2.2 Study Population

The study included all adults aged ≥ 65 years who were hospitalized in one of the VAHNSI hospitals, met the inclusion criteria, and had a laboratory‐confirmed diagnosis of RSV or influenza, including those with other viral co‐infections. Patients with co‐infections of RSV and influenza were excluded. Similarly, co‐infections involving both RSV subtypes were excluded when the objective was to compare the clinical severity of RSV A and B infections.

### 2.3 Study Period and Follow‐Up

This study spans six consecutive seasons, from 2014/15 to 2019/20. During the 2014/15 season, 10 hospitals participated in the VAHNSI network, while participation was limited to four hospitals in the subsequent seasons (2015/16 to 2019/20). Eligible cases were identified within the surveillance period defined as November 1 (epidemiological week 44) through March 31 (epidemiological week 13). Patients were followed from the date of hospital admission until 30 days post‐discharge, using data retrieved from the VID records.

### 2.4 Severity and Mortality Outcomes and Hospitalization Costs

The severity and mortality outcomes were use of MV (including pressure control ventilation, noninvasive positive pressure ventilation such as CPAP or BiPAP, high‐flow ventilation, and endotracheal intubation), hospital LOS in days, mortality during the hospitalization (in‐hospital death), and mortality during the hospital stay or within 30 days post‐discharge (day‐30 death). Data from VAHNSI network were used to identify MV and LOS, while in‐hospital death and day‐30 death were retrieved from VID.

Costs associated with hospitalizations were extracted from the hospital database from VID, where the Diagnosis Related Groups (DRG) system is used to assign each hospitalization episode to a degree of complexity. Official taxes from the specific season were used to assign a cost to each hospitalization (see Appendix S2 for a detailed description of the outcomes and costs).

### 2.5 Covariates

Key variables collected from VID included age at the admission, sex, vaccination status (hospitalized patients who had received the current season's influenza vaccine at least 15 days prior to admission were considered as vaccinated, patients hospitalized during the 15 days after vaccination were excluded when vaccination status was considered in the analysis, and patients without prior vaccination with the current season's influenza vaccine were considered as unvaccinated), and comorbidities (diabetes, chronic obstructive pulmonary disease [COPD], heart failure, and immunocompromised). To identify comorbidities, ICD codes were systematically searched in the ambulatory and hospitalization databases from VID during the study period (see Appendix S3 for ICD codes used to identify comorbidities in VID). A subject was considered to have the comorbidity if at least one corresponding diagnostic code was recorded on or before the hospital admission date at which the patient was recruited into the study.

### 2.6 Data Analysis

Demographics, clinical characteristics, influenza vaccination status, severity outcomes, cost, and seasonality of hospitalizations of enrolled patients were described by infecting virus (RSV and influenza) as well as by RSV subtype (A and B). Differences in patient characteristics and chronic conditions between RSV and influenza, and between RSV subtypes A and B, were tested using two‐sided chi‐square (categorical variables) and Student *t* tests (continuous variables); *p* value < 0.05 was considered statistically significant.

#### 2.6.1 Severity and Mortality Outcomes

Logistic regression models were used to compare the use of MV and death, and log‐normal regression models were used to compare the LOS of RSV hospitalizations with influenza hospitalizations. Similarly, severity outcomes and mortality were compared between RSV A‐ and RSV B‐associated hospitalizations. Odds ratios or mean ratios (ORs/MRs) and their 95% confidence intervals (CIs) were calculated. Models were adjusted by age, sex, season, influenza vaccination status, and comorbidities (see Appendix S2 in supplementary material for a more detailed description of the data analysis). In total, considering the study period without splitting by seasons, 18 patients had two admissions in the comparison between RSV and influenza‐vaccinated cases, and 12 patients had two admissions in the comparison between RSV and influenza‐unvaccinated cases, either with two influenza admissions or one influenza and one RSV admission. Clustered sandwich variance estimators were applied in all models to account for within‐patient correlation arising from multiple admissions.

#### 2.6.2 Cost of Hospitalizations

Costs associated to influenza, RSV, RSV A, and RSV B hospital admissions were described and compared through log‐normal regression models. Models were adjusted for age, sex, season, influenza vaccination status, and comorbidities. Only patients with complete costs data for models were analyzed. Clustered sandwich variance estimators were applied in all models to account for within‐patient correlation arising from multiple admissions. An additional analysis was conducted in which all observations deviating by more than 4 standard deviations from the logarithm of the mean cost were excluded.

#### 2.6.3 Sensitivity Analysis

To assess whether influenza vaccination was associated with reduced severity of influenza‐related hospitalizations compared with those caused by RSV in our study, a sensitivity analysis was conducted to estimate ORs and MRs for severity outcomes and hospitalization costs associated with RSV hospitalizations, comparing these with two subgroups of patients hospitalized with influenza: (1) those who had not received the current season's influenza vaccine (unvaccinated influenza hospitalizations) and (2) those who had received the vaccine at least 15 days prior to admission (vaccinated influenza hospitalizations). Demographics and clinical characteristics of vaccinated and unvaccinated patients were described, and differences were assessed as specified above.

## 3 Results

**FIGURE 1:**
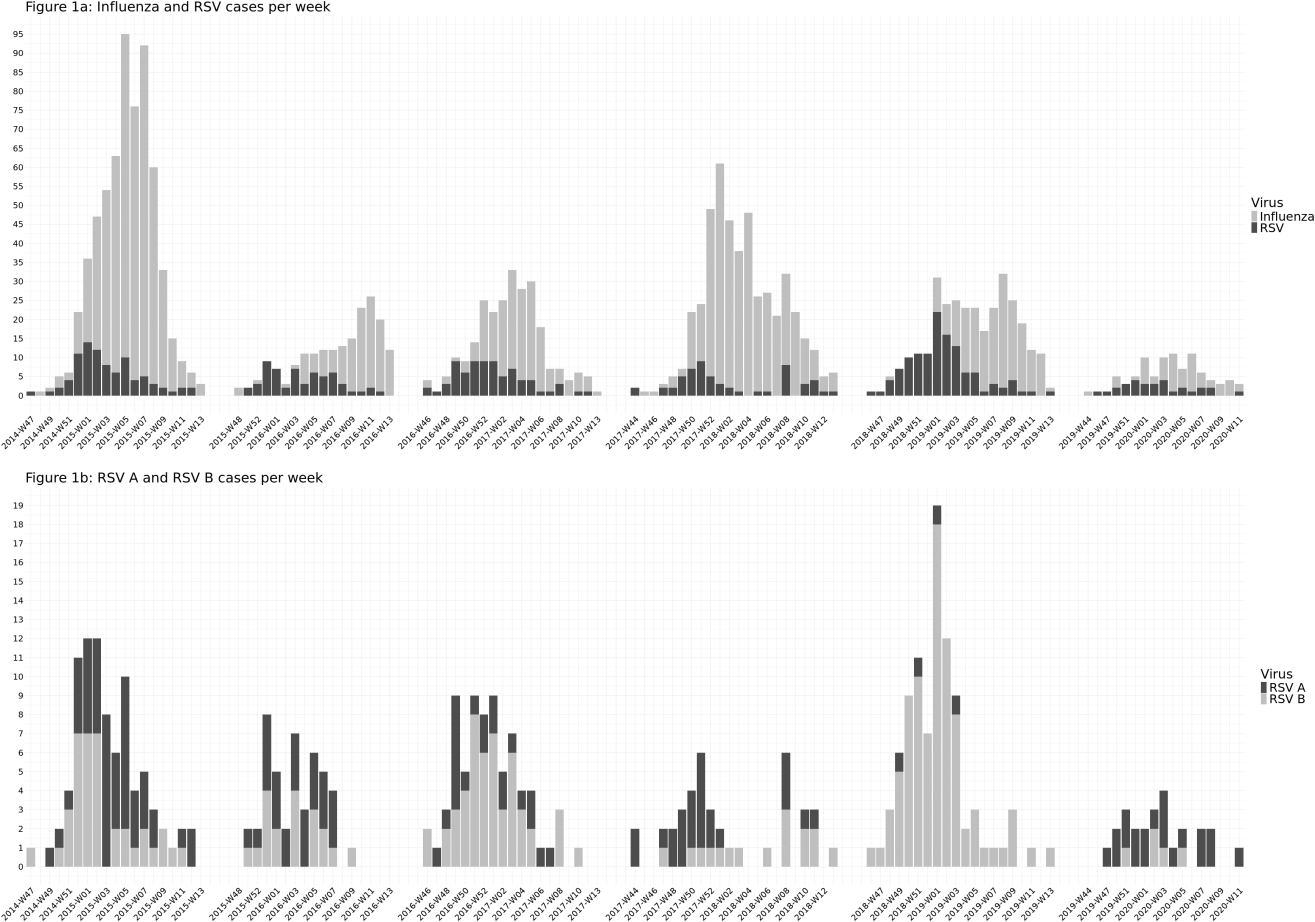
Weekly number of hospitalizations due to RSV or influenza (a), and RSV A or RSV B (b) virus infection in ≥ 65‐year‐olds included in the study.

In the 2014/15, 2015/16 and 2016/17 seasons, no records were available until approximately the second week of November (Figure 1). The last season, 2019/2020, ended in mid‐March due to the COVID‐19 pandemic. The number of patients aged ≥ 65 included in the study in each season ranged from 629 in 2014/15 to 90 in 2019/20 (Table 1). The first RSV and influenza hospitalizations appeared simultaneously in most seasons. However, RSV hospitalizations were more frequent during December and January, with particularly high prevalence in the 2018/2019 season, whereas most influenza hospitalizations occurred between January and March.

**TABLE 1:** Patient demographic and clinical characteristics and seasonality of influenza, respiratory syncytial virus (RSV), and RSV A and RSV B hospitalizations among adults aged ≥ 65 included in the study.

	Influenza, *N* = 1525	RSV, *N* = 429	*p*	RSV A, *N* = 146	RSV B, *N* = 215	*p*
Patient characteristics						
Sex			< 0.001			> 0.9
Female	757 (49.64%)	254 (59.21%)		85 (58.22%)	125 (58.14%)	
Male	768 (50.36%)	175 (40.79%)		61 (41.78%)	90 (41.86%)	
Age at the admission			< 0.001			0.5
Mean (SD)	80 (8)	82 (8)		82 (8)	82 (8)	
Median (IQR)	81 (74, 86)	83 (77, 88)		83 (77, 88)	82 (76, 87)	
Range	(65,103)	(65,102)		(65,100)	(65,102)	
Age group			< 0.001			0.6
65–79	730 (47.87%)	163 (38.00%)		55 (37.67%)	87 (40.47%)	
80+	795 (52.13%)	266 (62.00%)		91 (62.33%)	128 (59.53%)	
Chronic condition						
Diabetes	770 (50.49%)	212 (49.42%)	0.7	64 (43.84%)	111 (51.63%)	0.15
COPD	847 (55.54%)	271 (63.17%)	0.005	92 (63.01%)	137 (63.72%)	0.9
Heart failure	685 (44.92%)	242 (56.41%)	< 0.001	77 (52.74%)	126 (58.60%)	0.3
Immunocompromised	539 (35.34%)	140 (32.63%)	0.3	35 (23.97%)	72 (33.49%)	0.052
Influenza vaccination status			0.002			0.5
No	699 (45.84%)	158 (36.83%)		57 (39.04%)	76 (35.35%)	
Yes	818 (53.64%)	263 (61.31%)		87 (59.59%)	134 (62.33%)	
Seasonality						
Season			< 0.001			< 0.001
2014–2015	541 (35.48%)	88 (20.51%)		49 (33.56%)	38 (17.67%)	
2015–2016	132 (8.66%)	58 (13.52%)		27 (18.49%)	19 (8.84%)	
2016–2017	181 (11.87%)	75 (17.48%)		22 (15.07%)	50 (23.26%)	
2017–2018	419 (27.48%)	57 (13.29%)		24 (16.44%)	16 (7.44%)	
2018–2019	192 (12.59%)	121 (28.21%)		4 (2.74%)	87 (40.47%)	
2019–2020	60 (3.93%)	30 (6.99%)		20 (13.70%)	5 (2.33%)	
Month of the year			< 0.001			0.3
November	15 (0.98%)	18 (4.20%)		7 (4.79%)	10 (4.65%)	
December	133 (8.72%)	146 (34.03%)		48 (32.88%)	74 (34.42%)	
January	614 (40.26%)	174 (40.56%)		55 (37.67%)	94 (43.72%)	
February	535 (35.08%)	65 (15.15%)		29 (19.86%)	25 (11.63%)	
March	228 (14.95%)	26 (6.06%)		7 (4.79%)	12 (5.58%)	

Abbreviations: COPD, chronic obstructive pulmonary disease; IQR, interquartile range; *N*, number of patients; RSV, respiratory syncytial virus; SD, standard deviation.

A total of 1954 adults aged ≥ 65 years, hospitalized by a respiratory infection and included in VAHNSI, had laboratory‐confirmed RSV or influenza and were enrolled in the study. Among them, 1525 (78%) tested positive for influenza and 429 (22%) for RSV (Table 1). Co‐infections with both influenza and RSV were identified in eight admissions and were excluded. Co‐infections with other viruses were detected in 2% of influenza cases and 4% of RSV cases (see Appendix S4 for co‐infections description).

A higher proportion of patients hospitalized with RSV were female (59.21% vs. 49.64%; *p* < 0.001), older (median age: 82 vs. 80 years; *p* < 0.001), and more likely to have certain comorbidities compared with those with influenza, particularly chronic obstructive pulmonary disease (COPD: 63.17% vs. 55.54%; *p* = 0.005) and heart failure (56.41% vs. 44.92%; *p* < 0.001). In addition, the proportion of individuals vaccinated against influenza was higher among RSV patients (61.31% vs. 53.92%; *p* = 0.002) (Table 1).

### 3.1 Comparison of Severity Outcomes Between RSV and Influenza

In the adjusted analyses, the severity outcomes were similar or significantly higher among patients hospitalized with RSV compared with those hospitalized with influenza. The proportion of patients receiving MV with RSV (9.26%) was more than double that of patients with influenza (4.15%); adjusted OR (aOR) 2.43 (95% CI: 1.61–3.67) (Table 2). The mean LOS was 7.7 and 6.8 days for RSV and influenza hospitalizations, respectively; a 10% longer stay for RSV patients (adjusted MR [aMR] 1.10 [95% CI: 1.02–1.19]). The proportion of patients who died in the hospital was 5.46% for RSV and 4.88% for influenza; aOR 1.07 (95% CI: 0.63–1.80). The proportion of patients who died during hospitalization or within 30 days post‐discharge was 10.45% for RSV and 8.17% for influenza; aOR 1.27 [95% CI: 0.87–1.87]. Although death proportions were higher in RSV patients, the confidence intervals included the null value (see Appendix S5 for detailed adjustments by severity outcome).

**TABLE 2:** Odd ratios (ORs) or mean ratios (MRs), and 95% confidence intervals (95%CI) of severity outcomes and costs of respiratory syncytial virus–associated hospitalizations versus influenza‐associated hospitalizations.

	Patients, *N* (%)		RSV vs. Influenza
	RSV (*n* = 421)	Influenza (*n* = 1517)	Adjusted OR/MR* (95% CI)
Mechanical ventilation	39 (9.26%)	63 (4.15%)	2.43 (1.61, 3.67)
Length of stay (in days)			
Mean (SD) Median (IQR)	7.7 (6.9) 6.0 (4.0, 9.0)	6.8 (5.3) 6.0 (4.0, 8.0)	1.10 (1.02, 1.19)
In‐hospital death	23 (5.46%)	74 (4.88%)	1.07 (0.63, 1.80)
Day 30 death	44 (10.45%)	124 (8.17%)	1.27 (0.87, 1.87)
Hospitalization cost (€)			
Mean (SD) Median (IQR)	3907 (2065) 3616 (2808, 4544)	3806 (3878) 3434 (2542, 4472)	1.06 (1.01, 1.10)

Abbreviations: CI, confidence interval; IQR, interquartile range; MR, mean ratio; *N*, number of patients; OR, odds ratio; RSV, respiratory syncytial virus; SD, standard deviation.

* Odds ratios (ORs) were estimated for the comparison of mechanical ventilation and death and mean ratios (MRs) for the comparison of length of stay and cost of admission. Coinfections including RSV and influenza were excluded.

### 3.2 Comparison of Severity Outcomes Between RSV A and RSV B

Both RSV subtypes co‐circulated in all seasons. RSV A was more prevalent in the 2014/2015, 2015/2016, 2017/2018, and 2019/2020 seasons, while RSV B was more prevalent in the 2016/2017 and 2018/2019 seasons, during which nearly all RSV cases, except four, were RSV B (Table 1 and Figure 1).

Of the 361 patients with RSV subtype identified, 146 (40.4%) were Subtype A and 215 (59.6%) were Subtype B. Characteristics such as sex, age, or comorbidities did not differ significantly between RSV subtypes (Table 1).

The comparison of RSV B to RSV A suggested a potential increase in the odds of MV use in RSV B admissions (12.09% for RSV B vs. 6.85% for RSV A); however, the results were not statistically significant; aOR 1.85 (95% CI: 0.89–4.15) (Table 3). Likewise, no statistically significant differences were found in hospital LOS or mortality outcomes between the RSV subtypes.

**TABLE 3:** Odd ratios (ORs) or mean ratios (MRs) and 95% confidence intervals (95%CI) of severity outcomes and costs of respiratory syncytial virus A versus respiratory syncytial virus B–associated hospitalizations.

	Patients, *N* (%)		RSV A vs. RSV B
	RSV A (*n* = 146)	RSV B (*n* = 215)	Adjusted OR/MR^a^ (95% CI)
Mechanical ventilation	10 (6.85%)	26 (12.09%)	1.85 (0.89, 4.15)
Length of stay			
Mean (SD) Median (IQR)	7.4 (5.5) 7.0 (4.0, 9.0)	7.1 (5.4) 6.0 (4.0, 9.0)	1.00 (0.85, 1.19)
In‐hospital death	9 (6.16%)	13 (6.05%)	Not enough sample
Day 30 death	17 (11.64%)	20 (10.23%)	0.90 (0.46, 1.81)
Hospitalization cost (€)			
Mean (SD) Median (IQR)	3795 (1650) 3616 (2774, 4544)	3994 (2441) 3616 (2808, 4508)	1.03 (0.94, 1.13)

Abbreviations: CI, confidence interval; IQR, interquartile range; MR, mean ratio; OR, odds ratio; *N*, number of patients; RSV, respiratory syncytial virus; SD, standard deviation.

^a^ Odds ratios (ORs) were estimated for the comparison of mechanical ventilation and death and mean ratios (MRs) for the comparison of length of stay and cost of admission. Coinfections including RSV A and RSV B were excluded.

### 3.3 Hospitalization Costs

The median hospitalization cost per admission was €3616 for RSV and €3434 for influenza. Additionally, 75% of RSV admissions incurred costs exceeding €2808, while 75% of influenza admissions exceeded €2542 (Table 2). The aMRs indicated a 6% higher mean cost for RSV hospitalizations compared with influenza hospitalizations (aMR 1.06 [95% CI, 1.01, 1.10]). Regarding the range of values, the highest costs were outliers, with maximum costs representing extreme values. There was no statistically significant difference in costs between RSV A and RSV B hospitalizations (Table 3).

Observations deviating by more than 4 standard deviations from the logarithm of the mean cost were excluded in an additional analysis. A total of 10 observations were removed, representing 0.51% of all influenza and RSV hospitalizations (seven influenza admissions and three RSV admissions). The aMR remained unchanged after removing these outliers.

### 3.4 Sensitivity Analysis

A higher proportion of vaccinated influenza hospitalizations were male (53.30% vs. 46.92%; *p* < 0.005) and more frequently presented with certain comorbidities compared with those unvaccinated (Appendix S6). Differences were significant in the case of COPD, 59.66% in vaccinated versus 50.79% in unvaccinated (*p* < 0.001). The ORs and MRs for severity outcomes and costs of RSV hospitalizations, when compared separately with unvaccinated and vaccinated influenza hospitalizations, were not significantly different from the analysis that included all influenza hospitalizations (Appendix S7).

## 4 Discussion

Our analysis is based on a unique setting in Spain, involving active and systematic testing for multiple respiratory viruses in the same laboratory, along with longitudinal follow‐up of patients aged ≥ 65 years who were hospitalized in 4–10 hospitals over six consecutive seasons, thus providing a robust dataset. We show that RSV hospitalizations, while less frequent than those for influenza, were more severe and costly. Specifically, RSV hospitalizations required more than twice the proportion of MV and were associated with longer hospital stays and higher hospitalization costs, as compared with influenza. In addition, mortality rates during hospitalization and within 30 days post‐discharge were similar for both RSV and influenza hospitalizations, regardless of influenza vaccination status.

Falsey and colleagues were among the first to highlight the significant disease burden of RSV in older and high‐risk adults by direct comparison to influenza [2]. Consistent with their findings and subsequent studies [2, 15, 37], our results indicate that adults hospitalized with RSV were slightly older and had a higher prevalence of comorbidities, particularly cardiopulmonary diseases, compared with those hospitalized with influenza. However, after adjusting for comorbidities, we observed that severity outcomes for older adults with RSV were either comparable to or significantly worse than those for patients with influenza.

Several other studies have also assessed the impact of RSV compared with influenza, reinforcing these findings. Consistent with our results, Pastula et al., in a retrospective study, found that although RSV hospitalizations were less frequent than those due to influenza, they showed higher in‐hospital mortality, increased use of MV, and longer hospital stays [14]. Meanwhile, Ackerson et al. reported that RSV patients were older and had higher rates of comorbidities, and their adjusted analyses showed that RSV was associated with COPD exacerbation and increased 1‐year mortality compared with influenza [15].

These studies, along with others with similar objectives, have been conducted primarily using medical record databases routinely collected in clinical practice but not intended for research purposes [14, 15, 21, 26, 38]. These databases are typically based on routine clinical diagnoses and coding, which may introduce misclassification biases due to inconsistent diagnostic testing practices or the underdiagnoses of certain infections, such as RSV, resulting from a lack of disease awareness among clinicians and limited test availability [38, 39, 40]. In addition, they generally include only patients who are tested as part of standard care, potentially leading to selection bias and limiting the generalizability of the findings [41]. In contrast, data from respiratory infection surveillance networks such as ours, where all patients with respiratory symptoms undergo systematic PCR testing, provide a more accurate and unbiased comparison. Our approach ensures comprehensive case detection, reduces misclassification, and offers a more reliable assessment of disease burden and severity, enhancing the validity of the findings.

More recently, two studies have further contextualized RSV burden compared with influenza by incorporating vaccination status into their analyses [17, 18]. While the overall severity of RSV hospitalizations was similar to that of unvaccinated influenza and COVID‐19 patients, it was significantly higher than that of vaccinated patients [18]. In a unique study from Spain, Vega‐Piris et al. analyzed three post‐pandemic seasons, coinciding with the initiation of active RSV surveillance alongside COVID and influenza in their setting [17]. Our study complements their findings by leveraging VAHNSI's systematic respiratory virus testing, in place since 2010, and VID's linked clinical records to include death within 30 days after discharge. While Vega‐Piris et al. only reported a higher ICU admission risk for RSV compared with vaccinated influenza patients, we report here new information regarding the comparison of the use of MV, length of hospital stays, and costs between RSV and influenza hospitalizations.

Unlike these previous studies [17, 18], we did not find differences in severity outcomes, mortality, or costs when stratifying influenza‐related hospitalizations by vaccination status. In Spain, influenza vaccination is mainly recommended for older adults and individuals with chronic conditions, and coverage remains suboptimal. Consequently, vaccinated patients in our cohort were generally older and had more comorbidities than unvaccinated individuals, potentially introducing residual confounding. Although our models adjusted for measured comorbidities, unmeasured factors such as frailty or functional status may not have been fully accounted for. Despite differences in study populations, healthcare settings, and circulating virus strains, the substantial disease burden and severity associated with RSV underscore the need for increased awareness, improved preventive measures through the implementation of approved vaccines, and the development of new and effective treatments, particularly for high‐risk populations.

Overall, RSV hospitalizations were associated with slightly greater healthcare utilization than influenza, with a 10% longer mean LOS and 6% higher mean hospitalization costs. Although modest at the individual level, these differences may be relevant from a health system perspective, as small per‐admission increases can accumulate into substantial additional bed‐days and expenditures during peak respiratory virus seasons. These findings highlight the importance of considering both clinical outcomes and resource use when assessing the burden of RSV and the potential impact of preventive strategies.

We also contribute new data on the analysis of RSV subtypes. RSV A and B co‐circulated in all seasons, although one subtype often predominated each season. As noted by Surie et al. [18], limited data exist comparing the severity of RSV A and RSV B infections in adults. Our findings showed no significant differences in patient characteristics, such as sex, age, or comorbidities, between infections caused by RSV subtypes A and B. Regarding severity outcomes, RSV B admissions showed a potential increase in MV use, yet the results were not statistically significant. Overall, differences between RSV subtypes were not statistically significant, consistent with the limited published data [18, 29, 30, 31].

Our study has some limitations. First, while the higher prevalence of baseline cardiopulmonary disease in RSV patients likely contributed to worse clinical outcomes, these differences persisted even after adjusting for comorbidities and age. Second, in a recent study, we showed that using a case definition based on influenza‐like illness leads to an under‐detection of RSV cases in adults ranging from 13% to 40% [9]. Therefore, although some degree of RSV case under‐ascertainment cannot be completely ruled out, it is unlikely to have biased the comparative estimates of severity, mortality, or mean hospitalization costs between RSV and influenza cases, which was the objective of our study rather than estimating incidence or total case counts. Third, the premature termination of the 2019/2020 season due to the emergence of SARS‐CoV‐2 may have reduced the number of hospitalizations captured during the final weeks of surveillance—potentially affecting influenza cases to a greater extent, given that influenza generally circulates later than RSV—and, although this likely had a limited impact on comparative in‐hospital severity and cost estimates, some degree of bias related to changes in case mix or viral circulation patterns cannot be entirely excluded. Fourth, ICU admission could not be evaluated in our setting because data were not adequately collected due to limited access to ICU patients for obtaining informed consent. Finally, data from a 4–10‐hospital network in a single region may not be representative of the entire country. Although VAHNSI actively monitored and screened admissions, hospitalization criteria may vary across health departments. However, such differences likely affected RSV and influenza similarly, minimizing bias.

## 5 Conclusion

In summary, RSV hospitalizations in older adults were less frequent than those for influenza, but more severe, requiring greater use of MV and resulting in longer hospital stays and higher costs. Mortality rates were similar for both viruses. These findings underscore the importance of further developing therapeutic and preventive interventions against RSV infection in the elderly, and provide valuable evidence for designing and evaluating RSV vaccination strategies. From a policy perspective, prioritizing older adults—particularly those at higher risk of severe respiratory disease—could enhance the cost‐effectiveness profile of RSV vaccination programs by reducing high‐cost, high‐severity hospitalizations.

## Author Contributions

**Elisa Correcher‐Martínez:** writing – review and editing, methodology, formal analysis, data curation, conceptualization, software. **Cintia Muñoz‐Quiles:** conceptualization, writing – original draft, writing – review and editing, supervision, methodology, visualization. **Mónica López‐Lacort:** conceptualization, methodology, writing – review and editing, formal analysis, supervision, data curation. **Ainara Mira‐Iglesias:** writing – review and editing, conceptualization, methodology. **María Díaz‐López:** conceptualization, writing – review and editing, data curation. **Beatriz Mengual‐Chuliá:** investigation, writing – review and editing, resources. **F. Xavier López‐Labrador:** writing – review and editing, data curation, investigation, resources. **Javier Díez‐Domingo:** conceptualization, writing – review and editing, methodology. **Alejandro Orrico‐Sánchez:** conceptualization, funding acquisition, resources, writing – review and editing, project administration, methodology.

## Funding

This study was supported by a research grant from Investigator‐Initiated Studies Program of Merck Sharp & Dohme LLC and by the Instituto de Salud Carlos III (from the European funds of the Recovery, Transformation and Resilience Plan, file code CD22/00122, by virtue of the Resolution of the Directorate of the Instituto de Salud Carlos III, O.A., M.P., of 14 December 2022, awarding the Sara Borrell contracts of the 2022 call of the Strategic Action in Health 2021–2023), and the European Union—NextGenerationEU.

## Disclosure

The (partial) funder had no role in study design, data collection and analysis, decision to publish, or preparation of the manuscript. The opinions expressed in this paper are those of the authors and do not necessarily represent those of Merck Sharp & Dohme LLC.

## Ethics Statement

The study was conducted in accordance with Spanish legislation, institutional guidelines, the Declaration of Helsinki, and the International Conference on Harmonisation (ICH) Good Clinical Practice (GCP) guidelines. The protocol was approved by the Ethics Committee of the Hospital General Universitario de Elche. Written informed consent was obtained from all participants prior to their enrolment in the VAHNSI network. Additional data from various sources of VID were cross‐referenced using a previously dissociated personal identification number to ensure confidentiality.

## Conflicts of Interest

E.C.‐M. and B.M.‐C. declare no conflict of interest. M.L.‐L., C.M.‐Q., A.M.‐I., A.O.‐S., and J.D.‐D. have attended to several congresses whose registration, travel, and accommodation costs have been covered by pharmaceutical companies. J.D.‐D. has been principal investigator in clinical trials sponsored by SPMSD, MSD, GSK, and Pfizer. J.D.‐D. and A.O.‐S. acted as advisors for GSK and SPMSD. FXL‐L received grants from the Foundation for Influenza Epidemiology (France), payments to his institution from SP and from CIBERESP (Instituto de Salud Carlos III, Spain), and individual payments for advisory boards from SP.

## Supporting information


**Appendix S1:** Hospitals participating in the Valencia Hospital Surveillance Network for the Study of Influenza and Other Respiratory Viruses (VAHNSI).
**Appendix S2:** Data analysis.
**Appendix S3:** ICD codes systematically searched in the ambulatory and hospitalization databases from the Valencia Health System Integrated Database (VID) to identify populations with specific chronic conditions.
**Appendix S4:** Absolute and relative frequencies of viral co‐infections in Influenza and RSV cases.
**Appendix S5:** Detailed adjustments by severity outcome.
**Appendix S6:** Patient characteristics of influenza vaccinated and influenza unvaccinated hospitalizations among adults aged ≥ 65 included in the study.
**Appendix S7:** Sensitivity analysis: odd ratios (ORs) or mean ratios (MRs) and 95% confidence intervals (95%CI) of severity outcomes and costs of respiratory syncytial virus‐associated hospitalizations versus influenza‐associated hospitalizations by influenza vaccination status.

## Data Availability

Authors agree to make data and materials supporting the results presented in the paper available upon reasonable request.
