# Supplementary material for: Severity and Cost of Respiratory Syncytial Virus Versus Influenza in Hospitalized Adults in Spain
Source: Influenza Other Respir Viruses. 2026 Apr 22;20(4):e70254. doi: 10.1111/irv.70254 (PMC13102681; doi:10.1111/irv.70254)
Supplement: Supplementary file 1 — Appendix S1: Hospitals participating in the Valencia Hospital Surveillance Network for the Study of Influenza and Other Respiratory Viruses (VAHNSI). Appendix S2: Data analysis. Appendix S3: ICD codes systematically searched in the ambulatory and hospitalization databases from the Valencia Health System Integrated Database (VID) to identify populations with specific chronic conditions. Appendix S4: Absolute and relative frequencies of viral co‐infections in Influenza and RSV cases. Appendix S5: Detailed adjustments by severity outcome. Appendix S6: Patient characteristics of influenza vaccinated and influenza unvaccinated hospitalizations among adults aged ≥ 65 included in the study. Appendix S7: Sensitivity analysis: odd ratios (ORs) or mean ratios (MRs) and 95% confidence intervals (95%CI) of severity outcomes and costs of respiratory syncytial virus‐associated hospitalizations versus influenza‐associated hospitalizations by influenza vaccination status. [file IRV-20-e70254-s001.docx]

**Supplementary material**

**Appendix 1.** **Hospitals participating in the Valencia Hospital Surveillance Network for the Study of Influenza and Other Respiratory Viruses (VAHNSI).**

The study was conducted using data from the Valencia Hospital Surveillance Network for the Study of Influenza and Other Respiratory Viruses (VAHNSI) from the 2014/15 season to the 2019/20 season. During these seasons, between 4 and 10 of the following hospitals in the Valencia region of Spain participated in VAHNSI: Hospital General Universitario de Castellón (Castellón, Spain), Hospital Universitario de La Plana (Villarreal, Spain), Hospital Universitario y Politécnico La Fe (Valencia, Spain), Hospital Universitario Doctor Peset (Valencia, Spain), Hospital Universitario de La Ribera (Alzira, Spain), Hospital Lluís Alcanyís de Xàtiva (Xàtiva, Spain), Hospital Arnau de Vilanova (Valencia, Spain), Hospital Universitario San Juan de Alicante (San Juan de Alicante, Spain), Hospital General Universitario de Elda (Elda, Spain), Hospital General Universitario de Alicante (Alicante, Spain), and Hospital Universitario del Vinalopó (Elche, Spain).

| **Hospitals** | **2014/15** | **2015/16** | **2016/17** | **2017/18** | **2018/19** | **2019/20** |
| --- | --- | --- | --- | --- | --- | --- |
| General Universitario de Castellón | X | X | X | X | X | X |
| Universitario de La Plana | X |  |  |  |  |  |
| Arnau de Vilanova | X |  |  |  |  |  |
| Universitario y Politécnico La Fe | X | X | X | X | X | X |
| Universitario Doctor Peset | X | X | X | X | X | X |
| Universitario de La Ribera | X |  |  |  |  |  |
| Universitario San Juan de Alicante | X |  |  |  |  |  |
| General Universitario de Elda | X |  |  |  |  |  |
| General Universitario de Alicante | X | X | X | X | X | X |
| Universitario del Vinalopó | X |  |  |  |  |  |

**Appendix 2. Data analysis**

***Comparison of severity outcomes between RSV and influenza***

Severity outcomes were compared between RSV and influenza admissions. Previous studies have suggested that influenza vaccination may attenuate the severity of influenza-related hospitalizations. Consequently, RSV severity was comparable to unvaccinated influenza cases, but substantially higher than in vaccinated influenza patients. For this reason, we adjusted our models for influenza vaccination status as a confounder in the analysis of severity outcomes. Furthermore, we conducted a sensitivity analysis to estimate odds ratios (ORs) and mean ratios (MRs) for severity outcomes and hospitalization costs associated with RSV, comparing these with two subgroups of patients hospitalized with influenza: (1) those who had not received the seasonal influenza vaccine (unvaccinated), and (2) those who had received the vaccine at least 15 days prior to hospital admission (vaccinated).

These analyses were conducted without splitting by seasons, resulting in 18 patients being admitted twice during the study period in the RSV and influenza vaccinated group, and 12 patients being admitted twice in the RSV and unvaccinated group, with both influenza admissions or one an influenza admission and the other an RSV admission. Therefore, clustered sandwich variance estimators were used in all models to account for potential autocorrelation between multiple admissions from the same individual.

Mechanical ventilation (MV) includes any therapeutic strategy that consists of replacing or mechanically assisting spontaneous pulmonary ventilation when it is non-existent or ineffective for life (e.g., pressure control ventilation, non-invasive positive pressure ventilation, high-flow ventilation, CPAP, BiPAP, endotracheal intubation. Nasal goggles and Venturi mask are not considered as mechanical ventilation). The likelihood of MV use was compared between influenza and RSV admissions using logistic regression, with odds ratios (ORs) quantifying its association with virus type. The model was adjusted by sex, influenza vaccination status and age group. The presence of chronic conditions was not considered as an adjusting covariate because almost no patient without at least one chronic condition required MV.

Hospital length of stay (LOS), measured in days, was compared between RSV and influenza admissions using a log-normal regression model. This model assumes that the logarithm of LOS follows a normal distribution, which is appropriate given the positively skewed nature of LOS. The exponentiated coefficients from the model are interpreted as mean ratios (MRs), representing the multiplicative change in expected LOS between virus types. The model was adjusted by sex, age group, season, influenza vaccination status and presence of chronic conditions.

In-hospital and 30-day post-discharge mortality were compared between virus types using logistic regression, with odds ratios (ORs) estimating the likelihood of death associated with RSV versus influenza admissions. The regression was done adjusting by sex, age, influenza vaccination status, presence of chronic conditions and season. Season 2019-2020 was not included in this comparison due the low number of outcomes.

***Comparison of severity outcomes between RSV A and B***

Severity outcomes were compared between RSV A and RSV B. This analysis was performed without splitting the admissions by season, but there was no patient repeated across seasons.

The likelihood of MV use was compared between RSV A and RSV B admissions using logistic regression, with odds ratios (ORs) quantifying its association with virus subtype. The model was adjusted by sex and age group. The presence of chronic conditions was not considered as an adjusting covariate because almost no patient without at least one chronic condition required MV.

Hospital length of stay (LOS), measured in days, was compared between RSV A and RSV B admissions using a log-normal regression model. This model assumes that the logarithm of LOS follows a normal distribution, which is appropriate given the positively skewed nature of LOS. The exponentiated coefficients from the model are interpreted as mean ratios (MRs), representing the multiplicative change in expected LOS between virus subtypes. The model was adjusted by sex, age group, presence of chronic conditions and season.

In-hospital and 30-day post-discharge mortality were compared between virus subtypes using logistic regression, with odds ratios (ORs) estimating the likelihood of death associated with RSV A versus RSV B admissions. The regression was done adjusting by sex, age and presence of chronic conditions and without stratifying per season.

**Costs of hospitalizations**

Costs associated to the hospitalizations were extracted from the hospital database from VID, where Diagnosis Related Groups (DRG) system is used to assign each hospitalization episode to a degree of complexity. Depending on the season, All Patients Diagnosis Related Groups (AP DRGs, v27) or All Patients Refined Diagnosis Related Groups (APR DRGs, v32 and v35) were used (1–6). Patients are classified in homogeneous groups in terms of resource consumption (7). Hospital costs were analysed independently of the season in which income was generated; however, when allocating costs to DRGs, official taxes from the specific season were used.

Costs related with admissions, measured in euros, were compared using log normal regression to calculate MRs, which represented how the hospitalisation cost changes depending on the detected virus. The comparison was done adjusting by sex, age, vaccination status, presence of chronic conditions and season. Cases with missing cost data were excluded from this analysis (8 influenza-hospitalized patients).

An additional analysis was conducted, where all observations deviating more than 4 standard deviations from the logarithm of the mean cost were excluded.

*References*

1. Ley de Tasas de la Generalitat 5/2013, versión DRGs AP27 [Internet]. Disponible en: https://www.boe.es/boe/dias/2014/01/31/pdfs/BOE-A-2014-970.pdf

2. Ley de Tasas de la Generalitat 7/2014, versión DRGs AP27 [Internet]. Disponible en: https://dogv.gva.es/datos/2014/12/29/pdf/2014_11805.pdf

3. Ley de Tasas de la Generalitat 10/2015, versión DRGs AP27 [Internet]. Disponible en: https://www.boe.es/boe/dias/2016/02/08/pdfs/BOE-A-2016-1205.pdf

4. Ley de Tasas de la Generalitat 13/2016, versión APR32 [Internet]. Disponible en: https://www.boe.es/boe/dias/2017/02/09/pdfs/BOE-A-2017-1291.pdf

5. Ley de Tasas de la Generalitat 27/2018, versión APR35 [Internet]. Disponible en: https://dogv.gva.es/auto/dogv/docvpub/rlgv/2017/L_2017_20_ca_L_2018_27.pdf

6. Ley de Tasas de la Generalitat 10/2019, versión APR35 [Internet]. Disponible en: https://www.boe.es/boe/dias/2020/01/28/pdfs/BOE-A-2020-1204.pdf

7. de Miguel Díez J, López de Andrés A, Jiménez García R. El Conjunto Mínimo Básico de Datos (CMBD), nuestro big data para para la investigación epidemiológica de la patología respiratoria. Arch Bronconeumol [Internet]. 1 de mayo de 2020 [citado 28 de marzo de 2025];56(5):267-8. Disponible en: http://archbronconeumol.org/es-el-conjunto-minimo-basico-datos-articulo-S0300289619302327

**Appendix 3. ICD codes systematically searched in the ambulatory and hospitalization databases from the Valencia Health System Integrated Database (VID) to identify populations with specific chronic conditions.**

| *Chronic condition* | *ICD-9 CM* | *ICD-10 CM* |
| --- | --- | --- |
| Diabetes | 250.xx | E10-E14.xx |
| COPD | 490-496.xx | J44.0, J44.1, J43-J45.xx |
| Heart failure | 428-428.9 | I25.xx, I42.xx, I50.xx |
| Immunodeficiency  (including HIV, congenital/acquired immunodeficiency, malignant neoplasms and other immunodeficiency disorders and autoimmune diseases) | V08, 042, 079.53, 795.71  279.00-279.9  140.0-208.xx, 99.25, 99.28  242.00-242.01  245.2  288.00-288.9  340  357.0  358.00-358.01  555.0-555.9  556.0-556.9  710.0-710.9  714.00-714.9  696.x | Z21, B20-B24, B97.35, R75  D80-D89  C00-C97, Z51.1  E05.0  E06.3  D72  G35  G61.0  G70.0  K50  K51  M32, L93  M05, M06  L40, M07.0-M07.3, M09.0 |

Abbreviation: COPD, chronic obstructive pulmonary disease; HIV, human immunodeficiency virus; ICD-9 CM and ICD-10 CM, International Classification of Diseases, 9^th^ and 10^th^ Revision, Clinical Modification.

**Appendix 4. Absolute and relative frequencies of viral co-infections in Influenza and RSV cases**

| **Co-infecting virus** | **Influenza,** N = 1,517 | **RSV,** N = 421 |
| --- | --- | --- |
| No coinfection | 1486 (97.96%) | 405 (96.2%) |
| Adenovirus | 0 (0.00%) | 2 (0.48%) |
| Bocavirus | 2 (0.13%) | 1 (0.24%) |
| Coronavirus | 14 (0.92%) | 4 (0.95%) |
| Metapneumovirus | 5 (0.33%) | 1 (0.24%) |
| Rhino/Enterovirus | 7 (0.46%) | 7 (1.66%) |
| Rhino/Enterovirus + Bocavirus | 0 (0.00%) | 1 (0.24%) |
| Rhino/Enterovirus + Coronavirus | 3 (0.2%) | 0 (0.00%) |

Abbreviation: N, Number of patients; RSV, respiratory syncytial virus.

**Appendix 5. Detailed adjustments by severity outcome.**

Use of **mechanical ventilation** adjusted by test result, vaccination status, sex and age group.

|  | **Adjusted OR** | **CI 95%** |
| --- | --- | --- |
| **Virus** |  |  |
| Influenza | Reference | - |
| RSV | 2.43 | 1.61, 3.67 |
| **Vaccinated** |  |  |
| No | Reference | - |
| Yes | 1.12 | 0.73, 1.71 |
| **Sex** |  |  |
| Female | Reference | - |
| Male | 1.15 | 0.75, 1.75 |
| **Age group** |  |  |
| 65-79 | Reference | - |
| +80 | 0.79 | 0.52, 1.20 |

Use of **mechanical ventilation** adjusted by test result, sex and age group.

|  | **Adjusted OR** | **CI 95%** |
| --- | --- | --- |
| **Virus** |  |  |
| Influenza (not vaccinated) | Reference | - |
| Influenza (vaccinated) | 1.22 | 0.73, 2.04 |
| RSV | 2.83 | 1.72, 4.67 |
| **Sex** |  |  |
| Female | Reference | - |
| Male | 1.15 | 0.76, 1.76 |
| **Age group** |  |  |
| 65-79 | Reference | - |
| +80 | 0.79 | 0.52, 1.20 |

**Length of stay** adjusted by test result, vaccination status, sex, age group, chronic conditions and season.

|  | **Adjusted MR** | **CI 95%** |
| --- | --- | --- |
| **Virus** |  |  |
| Influenza | Reference | - |
| RSV | 1.10 | 1.02, 1.19 |
| **Vaccinated** |  |  |
| No | Reference | - |
| Yes | 0.98 | 0.92, 1.04 |
| **Sex** |  |  |
| Female | Reference | - |
| Male | 0.96 | 0.90, 1.02 |
| **Age group** |  |  |
| 65-79 | Reference | - |
| +80 | 1.03 | 0.97, 1.09 |
| **Chronic conditions** |  |  |
| No | Reference | - |
| Yes | 1.10 | 1.00, 1.21 |
| **Season** |  |  |
| 14-15 | Reference | - |
| 15-16 | 1.07 | 0.95, 1.20 |
| 16-17 | 0.91 | 0.83, 1.00 |
| 17-18 | 1.05 | 0.97, 1.13 |
| 18-19 | 0.97 | 0.88, 1.08 |
| 19-20 | 0.99 | 0.85, 1.15 |

**Length of stay** adjusted by test result, sex, age group, chronic conditions and season.

|  | **Adjusted MR** | **CI 95%** |
| --- | --- | --- |
| **Virus** |  |  |
| Influenza (not vaccinated) | Reference | - |
| Influenza (vaccinated) | 1.00 | 0.93, 1.07 |
| RSV | 1.09 | 1.01, 1.19 |
| **Sex** |  |  |
| Female | Reference | - |
| Male | 0.96 | 0.90, 1.02 |
| **Age group** |  |  |
| 65-79 | Reference | - |
| +80 | 1.02 | 0.96, 1.09 |
| **Chronic conditions** |  |  |
| No | Reference | - |
| Yes | 1.09 | 1.00, 1.20 |
| **Season** |  |  |
| 14-15 | Reference | - |
| 15-16 | 1.07 | 0.95, 1.20 |
| 16-17 | 0.91 | 0.83, 1.00 |
| 17-18 | 1.05 | 0.97, 1.14 |
| 18-19 | 0.98 | 0.88, 1.08 |
| 19-20 | 0.99 | 0.85, 1.15 |

**In-hospital death** adjusted by test result, vaccination status, sex, age group, chronic conditions and season.

|  | **Adjusted OR** | **CI 95%** |
| --- | --- | --- |
| **Virus** |  |  |
| Influenza | Reference | - |
| RSV | 1.07 | 0.63, 1.80 |
| **Vaccinated** |  |  |
| No | Reference | - |
| Yes | 0.87 | 0.57, 1.35 |
| **Sex** |  |  |
| Female | Reference | - |
| Male | 0.76 | 0.48, 1.19 |
| **Age group** |  |  |
| 65-79 | Reference | - |
| +80 | 3.13 | 1.86, 5.25 |
| **Chronic conditions** |  |  |
| No | Reference | - |
| Yes | 0.59 | 0.35, 1.02 |
| **Season** |  |  |
| 14-15 | Reference | - |
| 15-16 | 1.93 | 0.99, 3.76 |
| 16-17 | 1.26 | 0.65, 2.42 |
| 17-18 | 1.17 | 0.66, 2.06 |
| 18-19 | 0.94 | 0.47, 1.89 |

**In-hospital death** adjusted by test result, sex, age group, chronic conditions and season.

|  | **Adjusted OR** | **CI 95%** |
| --- | --- | --- |
| **Virus** |  |  |
| Influenza (not vaccinated) | Reference | - |
| Influenza (vaccinated) | 1.05 | 0.64, 1.72 |
| RSV | 1.07 | 0.60, 1.90 |
| **Sex** |  |  |
| Female | Reference | - |
| Male | 0.74 | 0.47, 1.17 |
| **Age group** |  |  |
| 65-79 | Reference | - |
| +80 | 3.12 | 1.86, 5.25 |
| **Chronic condition** |  |  |
| No | Reference | - |
| Yes | 0.58 | 0.34, 1.00 |
| **Season** |  |  |
| 14-15 | Reference | - |
| 15-16 | 1.96 | 1.00, 3.83 |
| 16-17 | 1.26 | 0.65, 2.43 |
| 17-18 | 1.19 | 0.67, 2.10 |
| 18-19 | 0.95 | 0.47, 1.91 |

**In-hospital death** adjusted by test result, sex, age group, chronic conditions and season.

|  | **Adjusted OR** | **CI 95%** |
| --- | --- | --- |
| **Virus** |  |  |
| Influenza (vaccinated) | Reference | - |
| Influenza (not vaccinated) | 0.95 | 0.58, 1.55 |
| RSV | 1.01 | 0.58, 1.78 |
| **Sex** |  |  |
| Female | Reference | - |
| Male | 0.74 | 0.47, 1.17 |
| **Age group** |  |  |
| 65-79 | Reference | - |
| +80 | 3.12 | 1.86, 5.25 |
| **Chronic condition** |  |  |
| No | Reference | - |
| Yes | 0.58 | 0.34, 1.00 |
| **Season** |  |  |
| 14-15 | Reference | - |
| 15-16 | 1.96 | 1.00, 3.83 |
| 16-17 | 1.26 | 0.65, 2.43 |
| 17-18 | 1.19 | 0.67, 2.10 |
| 18-19 | 0.95 | 0.47, 1.91 |

**Day 30 death** adjusted by test result, vaccination status, sex, age group, chronic conditions and season.

|  | **Adjusted OR** | **CI 95%** |
| --- | --- | --- |
| **Virus** |  |  |
| Influenza | Reference | - |
| RSV | 1.27 | 0.87, 1.87 |
| **Vaccinated** |  |  |
| No | Reference | - |
| Yes | 0.94 | 0.67, 1.31 |
| **Sex** |  |  |
| Female | Reference | - |
| Male | 0.74 | 0.52, 1.04 |
| **Age group** |  |  |
| 65-79 | Reference | - |
| +80 | 2.85 | 1.94, 4.18 |
| **Chronic conditions** |  |  |
| No | Reference | - |
| Yes | 0.73 | 0.47, 1.13 |
| **Season** |  |  |
| 14-15 | Reference | - |
| 15-16 | 1.45 | 0.84, 2.51 |
| 16-17 | 1.22 | 0.73, 2.05 |
| 17-18 | 1.20 | 0.78, 1.85 |
| 18-19 | 0.97 | 0.58, 1.62 |

**Day 30 death** adjusted by test result, sex, age group, chronic conditions and season.

|  | **Adjusted OR** | **CI 95%** |
| --- | --- | --- |
| **Virus** |  |  |
| Influenza (not vaccinated) | Reference | - |
| Influenza (vaccinated) | 0.99 | 0.68, 1.46 |
| RSV | 1.27 | 0.82, 1.96 |
| **Sex** |  |  |
| Female | Reference | - |
| Male | 0.73 | 0.52, 1.03 |
| **Age group** |  |  |
| 65-79 | Reference | - |
| +80 | 2.87 | 1.96, 4.21 |
| **Chronic condition** |  |  |
| No | Reference | - |
| Yes | 0.73 | 0.47, 1.13 |
| **Season** |  |  |
| 14-15 | Reference | - |
| 15-16 | 1.45 | 0.84, 2.52 |
| 16-17 | 1.27 | 0.76, 2.11 |
| 17-18 | 1.20 | 0.78, 1.86 |
| 18-19 | 0.96 | 0.57, 1.61 |

**Hospitalization costs** adjusted by test result, vaccination status, sex, age group, chronic conditions and season.

|  |  | | **Sensitivity analysis** | |
| --- | --- | --- | --- | --- |
|  | **Adjusted MR** | **CI 95%** | **Adjusted MR** | **CI 95%** |
| **Virus** |  |  |  |  |
| Influenza | Reference | - | Reference | - |
| RSV | 1.06 | 1.01, 1.10 | 1.06 | 1.02, 1.10 |
| **Vaccinated** |  |  |  |  |
| No | Reference | - | Reference | - |
| Yes | 0.97 | 0.94, 1.00 | 0.97 | 0.94, 1.00 |
| **Sex** |  |  |  |  |
| Female | Reference | - | Reference | - |
| Male | 1.02 | 0.99, 1.06 | 1.04 | 1.01, 1.07 |
| **Age group** |  |  |  |  |
| 65-79 | Reference | - | Reference | - |
| +80 | 1.03 | 1.00, 1.07 | 1.04 | 1.01, 1.08 |
| **Chronic conditions** |  |  |  |  |
| No | Reference | - | Reference | - |
| Yes | 1.07 | 1.01, 1.13 | 1.06 | 1.00, 1.11 |
| **Season** |  |  |  |  |
| 14-15 | Reference | - | Reference | - |
| 15-16 | 0.99 | 0.92, 1.06 | 0.97 | 0.91, 1.03 |
| 16-17 | 0.95 | 0.90, 1.00 | 0.96 | 0.91, 1.01 |
| 17-18 | 0.99 | 0.95, 1.03 | 0.99 | 0.95, 1.03 |
| 18-19 | 0.97 | 0.92, 1.03 | 0.97 | 0.93, 1.02 |
| 19-20 | 0.94 | 0.86, 1.03 | 0.92 | 0.86, 0.98 |

**Hospitalization costs** adjusted by test result, sex, age group, chronic condition and season.

|  |  | | **Sensitivity analysis** | |
| --- | --- | --- | --- | --- |
|  | **Adjusted MR** | **CI 95%** | **Adjusted MR** | **CI 95%** |
| **Virus** |  |  |  |  |
| Influenza (not vaccinated) | Reference | - | Reference | - |
| Influenza (vaccinated) | 0.98 | 0.95, 1.02 | 0.98 | 0.95, 1.02 |
| RSV | 1.04 | 0.99, 1.09 | 1.05 | 1.00, 1.09 |
| **Sex** |  |  |  |  |
| Female | Reference | - | Reference | - |
| Male | 1.02 | 0.99, 1.06 | 1.04 | 1.01, 1.07 |
| **Age group** |  |  |  |  |
| 65-79 | Reference | - | Reference | - |
| +80 | 1.03 | 1.00, 1.07 | 1.04 | 1.01, 1.08 |
| **Chronic conditions** |  |  |  |  |
| No | Reference | - | Reference | - |
| Yes | 1.07 | 1.02, 1.13 | 1.06 | 1.00, 1.11 |
| **Season** |  |  |  |  |
| 14-15 | Reference | - | Reference | - |
| 15-16 | 0.99 | 0.92, 1.06 | 0.97 | 0.91, 1.03 |
| 16-17 | 0.95 | 0.90, 1.00 | 0.96 | 0.91, 1.01 |
| 17-18 | 0.99 | 0.95, 1.03 | 0.99 | 0.95, 1.03 |
| 18-19 | 0.98 | 0.93, 1.03 | 0.98 | 0.93, 1.02 |
| 19-20 | 0.94 | 0.86, 1.03 | 0.92 | 0.86, 0.99 |

Abbreviation: RSV, respiratory syncytial virus; OR, odds ratio; MR, mean ratio.

**Appendix 6. Patient characteristics of influenza vaccinated and influenza unvaccinated hospitalizations among adults aged ≥65 included in the study.**

|  | **Influenza not vaccinated,**  N = 699 | **Influenza vaccinated,**  N = 818 | **p-value** |
| --- | --- | --- | --- |
| **Patient Characteristics** |  |  |  |
| **Sex** |  |  | 0.013 |
| Female | 371 (53.1%) | 382 (46.7%) |  |
| Male | 328 (46.9%) | 436 (53.3%) |  |
| **Age at the admission** |  |  | 0.2 |
| Mean (SD) | 80 (8) | 81 (8) |  |
| Median (Q1, Q3) | 80 (73, 86) | 81 (75, 86) |  |
| (Min,Max) | (65,101) | (65,103) |  |
| **Age group** |  |  | 0.7 |
| 65-79 | 339 (48.5%) | 389 (47.6%) |  |
| 80+ | 360 (51.5%) | 429 (52.4%) |  |
| **Chronic condition** |  |  |  |
| Diabetes | 345 (49.4%) | 421 (51.5%) | 0.4 |
| COPD | 355 (50.8%) | 488 (59.7%) | <0.001 |
| Heart failure | 300 (42.9%) | 380 (46.5%) | 0.2 |
| Immunocompromised | 239 (34.2%) | 296 (36.2%) | 0.4 |

Abbreviation: N, Number of patients; RSV, respiratory syncytial virus; SD, standard deviation; IQR, interquartile range; COPD, chronic obstructive pulmonary disease.

**Appendix 7. Sensitivity analysis: Odd Ratios (ORs) or Mean Ratios (MRs) and 95% Confidence Intervals (95%CI) of severity outcomes and costs of Respiratory Syncytial Virus-associated hospitalizations *vs.* influenza-associated hospitalizations, by influenza vaccination status.**

|  | **Patients, N (%)** | | | **RSV vs Influenza by vaccination status** | |
| --- | --- | --- | --- | --- | --- |
|  | **RSV (n= 429)** | **Influenza** | | **Unvaccinated**  **Adjusted OR/MR***  **(95% CI)** | **Vaccinated**  **Adjusted OR/MR***  **(95%CI)** |
|  |  | **Unvaccinated**  **(n = 699)** | **Vaccinated**  **(n = 818)** |  |  |
| **Mechanical ventilation** | 41 (9.56%) | 26 (3.72%) | 37 (4.52%) | 2.83 (1.72, 4.67) | 2.32 (1.47, 3.69) |
| **Length of stay (in days)**  Mean (SD)  Median (IQR) | 7.7 (6.9)  6.0 (4.0, 9.0) | 6.77 (5.34)  6.0 (3.0, 8.0) | 6.83 (5.31)  5.0 (4.0, 8.0) | 1.09 (1.01, 1.19) | 1.10 (1.01, 1.19) |
| **In-hospital death** | 23 (5.36%) | 35 (5.01%) | 39 (4.77%) | 1.07 (0.60, 1.90) | 1.01 (0.58, 1.78) |
| **Day 30 death** | 45 (10.49%) | 59 (8.44%) | 65 (7.95%) | 1.27 (0.82, 1.96) | 1.28 (0.84, 1.94) |
| **Hospitalization cost (€)**  Mean (SD)  Median (IQR) | 3,897 (2,049)  3,616 (2,808, 4,544) | 3,802 (2,848)  3,434 (2,542, 4,472) | 3,810 (4,577)  3,434 (2,542, 4,525) | 1.04 (0.99, 1.09) | 1.06 (1.01, 1.11) |

Abbreviation: N, Number of patients; RSV, respiratory syncytial virus; SD, standard deviation; IQR, interquartile range; OR, Odds Ratio; MR, Mean Ratio; CI, Confidence Interval.

*Odds ratios (ORs) were estimated for the comparison of mechanical ventilation and death and mean ratios (MRs) for the comparison of length of stay and cost of admission. Coinfections including RSV and influenza were excluded.
